# Evidence for adjustable bandwidth orientation channels

**DOI:** 10.3389/fpsyg.2014.00578

**Published:** 2014-06-12

**Authors:** Christopher P. Taylor, Patrick J. Bennett, Allison B. Sekuler

**Affiliations:** ^1^Department of Psychology and Clinical Language Sciences, Centre for Integrative Neuroscience and Neurodynamics, University of ReadingReading, UK; ^2^Department of Psychology, Neuroscience, and Behaviour, McMaster UniversityHamilton, ON, Canada; ^3^Centre for Vision Research, York UniversityToronto, ON, Canada

**Keywords:** pattern vision, orientation, channels, ideal observer, classification images, summation, psychophysics

## Abstract

The standard model of early vision claims that orientation and spatial frequency are encoded with multiple, quasi-independent channels that have fixed spatial frequency and orientation bandwidths. The standard model was developed using detection and discrimination data collected from experiments that used deterministic patterns such as Gabor patches and gratings used as stimuli. However, detection data from experiments using noise as a stimulus suggests that the visual system may use adjustable-bandwidth, rather than fixed-bandwidth, channels. In our previous work, we used classification images as a key piece of evidence against the hypothesis that pattern detection is based on the responses of channels with an adjustable spatial frequency bandwidth. Here we tested the hypothesis that channels with adjustable orientation bandwidths are used to detect two-dimensional, filtered noise targets that varied in orientation bandwidth and were presented in white noise. Consistent with our previous work that examined spatial frequency bandwidth, we found that detection thresholds were consistent with the hypothesis that observers sum information across a broad range of orientations nearly optimally: absolute efficiency for stimulus detection was 20–30% and approximately constant across a wide range of orientation bandwidths. Unlike what we found with spatial frequency bandwidth, the results of our classification image experiment were consistent with the hypothesis that the orientation bandwidth of internal filters were adjustable. Thus, for orientation summation, both detection thresholds and classification images support the adjustable channels hypothesis. Classification images also revealed hallmarks of inhibition or suppression from uninformative spatial frequencies and/or orientations. This work highlights the limitations of the standard model of summation for orientation. The standard model of orientation summation and tuning was chiefly developed with narrow-band stimuli that were not presented in noise, stimuli that are arguably less naturalistic than the variable bandwidth stimuli presented in noise used in our experiments. Finally, the disagreement between the results from our experiments on spatial frequency summation with the data presented in this paper suggests that orientation may be encoded more flexibly than spatial frequency channels.

## 1. Introduction

Visual noise has been used to investigate visual processing in a variety of tasks (Pelli and Farell, 1999). Researchers have used two-dimensional luminance noise most frequently, but studies have also used one-dimensional luminance noise (i.e., noise that is constrained to vary along a single dimension) as well as visual noise that varies in other ways such as color (Gegenfurtner and Kiper, 1992), motion (Dakin et al., 2005), orientation (Girshick et al., 2011), Gaussian spatial windowing or bubbles (Gosselin and Schyns, 2001), and zero-dimensional noise pedestal increments (Baker and Meese, 2012). In virtually all of these studies, the noise was used as a mask and the observer's task was to ignore the noise to detect a non-noise target. Comparatively few studies have used noise as the target stimulus itself. David Green and colleagues used noise in this way to study the mechanisms underlying the detection of auditory signals (Green, 1960a,b; Green and Swets, 1966), and subsequent studies adopted Green's approach to study vision (for examples, see(Kersten, 1987; Taylor et al., 2003, 2004, 2005, 2006, 2009; Levi et al., 2005, 2008). In the current study, we use noise targets and noise masks to investigate orientation selectivity of visual mechanisms.

Data from detection, discrimination, and adaptation experiments using both psychophysical and physiological methods support the idea that the early stages of visual processing encode patterns with channels that are tuned to a fixed range of spatial frequency and orientation (Campbell and Kulikowski, 1966; Campbell et al., 1966; Graham, 1989; Wandell, 1995). This standard, or back-pocket, model of early visual coding accounts for a wide range of detection and discrimination data (Klein, 1992; Watson, 2000). However, despite its many successes, the standard multiple channels model apparently fails to account for some experimental results (see Nachmias et al., 1973; Kersten, 1987; Derrington and Henning, 1989; Perkins and Landy, 1991; Wandell, 1995; Taylor et al., 2009). One particularly puzzling result was reported by Kersten (1987), who measured detection thresholds for a visual noise target embedded in a visual noise mask. Detection thresholds were measured with noise targets with different spatial frequency bandwidths. For frequency bandwidths between 1 and 4 octaves, Kersten found that detection thresholds were proportional to the quarter-root of bandwidth,

(1) $$c_{rms}\propto {\text{BW}}^{\frac{1}{4}}$$

Interestingly, Kersten showed that detection thresholds for an ideal observer also were proportional to the quarter-root of bandwidth, which implies that absolute efficiency (*η*), defined as

(2) $$\eta ={\left(\frac{c_{rms\;(ideal)}}{c_{rms\;(observer)}}\right)}^{2}$$

ought to be constant. Indeed, Kersten found that absolute efficiency was high (≈50%) and approximately constant as the spatial frequency bandwidth of the noise stimulus was increased from 0.5 to 4 octaves. Kersten pointed out that this result is surprising because ideal observers integrate information across the entire spatial frequency bandwidth, whereas human observers are thought to detect patterns using mechanisms that have bandwidths that are much narrower than four octaves. Hence, the data suggest that spatial frequency summation is approximately optimal across a wide bandwidth, and appear to be inconsistent with a standard model that assumes that patterns are detected using channels that have a fixed and relatively narrow frequency bandwidth. Instead, Kersten suggested that the data were consistent with the adjustable channels hypothesis, first proposed by Green (1960a,b) to explain similar results obtained in an auditory detection task, which states that human observers detect band-limited noise using a channel, or combination of channels, with a frequency bandwidth that is adjusted to match that of the stimulus, and which sums information efficiently across the entire bandwidth.

Taylor et al. (2009) evaluated the adjustable channels hypothesis by using the classification image technique (Murray, 2011) to measure the frequencies observers use to detect visual noise that varied in bandwidth from 0.5 to 6 octaves. Like Kersten (1987), Taylor et al. found that detection thresholds were proportional to the quarter-root of bandwidth (Equation 1). However, contrary to the predictions of the adjustable channels hypothesis, estimates of the spatial frequency bandwidth of the channel used to detect visual noise, which was derived from the classification image data, did not vary with stimulus bandwidth. Furthermore, Taylor et al. used Monte Carlo simulations to demonstrate that the optimal spatial frequency summation found in noise detection tasks was, surprisingly, consistent with the predictions of at least one version of the standard model (Wilson et al., 1983). In short, Taylor et al. showed that the apparently anomalous results reported by Kersten (1987) were consistent with standard models of spatial frequency summation (Graham, 1989).

In this paper, we follow up on our previous work on spatial frequency summation and investigate orientation summation in two experiments. The first experiment measures detection thresholds and absolute efficiency of noise patterns that vary in orientation bandwidth. The second experiment uses the classification image technique to estimate the tuning characteristics of the internal filters used in this noise detection task. To anticipate our results, we find that orientation summation is, like spatial frequency summation, nearly optimal across a wide range of bandwidths. However, unlike what was found with frequency summation, the classification image results are consistent with the hypothesis that the orientation bandwidth of the internal filter that mediates detection is adjusted to match the stimulus.

## 2. Experiment 1

### 2.1. Materials and methods

#### 2.1.1. Observers

The three observers (all female; 23–27 years of age) in this experiment were members of the McMaster University community and were paid for their participation. Informed consent was obtained from all participants and the research approved by the McMaster University research ethics board. All observers were naïve about the experimental hypotheses, had normal or corrected-to-normal Snellen acuity and Pelli–Robson contrast sensitivity, and had extensive practice with this and other visual psychophysical tasks.

#### 2.1.2. Apparatus

Stimuli were generated and displayed using an Apple Macintosh G4 computer with an ATI Radeon video card running MATLAB and the Psychophysics and Video toolboxes (Brainard, 1997; Pelli, 1997). The stimulus display was a Sony GDM-F520 monitor set to a resolution of 1024 × 768 pixels and subtended a visual angle of 10.8° × 8.3° at the viewing distance of 2 m. The frame rate of the display was 75 Hz and the mean luminance 45 cd/m^2^. Display luminance was calibrated using a PhotoResearch PR-650 photometer before each session. The results of the calibration were used to linearize the display for that session, but in general there was little variability in the display from session to session. A Cambridge Research System Bits++ device was used to achieve fine grained (i.e., 14-bit) control of contrast. A custom designed button box with an ActiveWire card was used to record the observer's responses.

#### 2.1.3. Stimuli

The stimuli were two-dimensional Gaussian white noise patterns that were spatially filtered digitally with ideal (hard-edged) spatial frequency and orientation filters. The filter had a fixed center spatial frequency of 5 cy/deg, but depending on the experimental condition the spatial frequency bandwidth was either one or two octaves. The center orientation of the filter was horizontal. The two-sided orientation bandwidths were 2°, 8°, 16°, 32°, 64°, 128°, and 180°. For example, a two-sided orientation bandwidth filter of 16° passed orientations from −8° to +8°. To prevent edge artifacts, stimulus contrast was modulated on the screen with a circularly-symmetric Gaussian envelope with a standard deviation of 1.08° of visual angle. A white noise mask with a contrast variance of 0.32 was used in all conditions to mask the signal noise. On each trial, a new sample of signal noise and background noise were generated on each interval on every trial. The monitor provided the only illumination in the testing room.

#### 2.1.4. Procedure

Observers viewed the stimuli binocularly through natural pupils. A two-interval forced-choice (2-IFC) procedure was used. The observer was instructed to fixate a high-contrast dot located in the center of the display. The observer initiated each trial by pressing the space-bar on the keyboard. After a delay of 50 ms, the fixation point was removed, then after another 50 ms delay the first stimulus interval appeared. The first stimulus interval was 200 ms in duration and was followed by a 300 ms blank inter-stimulus interval and then a second 200 ms stimulus interval. The two stimulus intervals were marked by clearly audible tones, and a high/low pitched tone indicated whether a response was correct/incorrect. The observer's task was to determine which of the two stimulus intervals contained the target.

Stimulus contrast variance was varied across trials using four interleaved staircases, two converging on the 71% correct point of the psychometric function and two on the 84% correct point (Wetherill and Levitt, 1965). The staircases were stopped when the observer had completed 75 trials in each staircase. The total number of trials in each session was 2100 (300 trials per stimulus bandwidth, and seven stimulus bandwidths/session). Thresholds, defined as the RMS contrast required to produce 75% correct, were estimated by fitting a cumulative normal to all the data collected.

A 3° × 3° square, drawn with a high-contrast, 2-pixel wide line, was centered on the fixation point surrounded the stimulus to reduce spatial uncertainty. The frame remained on the screen for the entire duration of each trial: it was centered on the fixation point at the start of a trial, and remained visible until the observer made a response. To reduce adaptation, the square had a 50% probability of being black or white on each trial.

Thresholds were measured with stimuli that had spatial frequency bandwidths of 1 or 2 octaves. Two spatial frequency bandwidth conditions were run in separate sessions, alternating with each session, and each observer began with a spatial frequency bandwidth chosen randomly. In each test session, orientation bandwidths were presented in separate blocks of trials and the order of bandwidths was randomized. All orientation bandwidth conditions were completed during a single session.

In addition to the conditions described above, we measured contrast detection thresholds for a white noise stimulus as control in a separate session for the same observers. The three observers completed four white noise detection sessions, each containing 300 trials. The thresholds from this control condition were collected after all other conditions in Experiment 1 were completed.

### 2.2. Results

**Figure 1:**
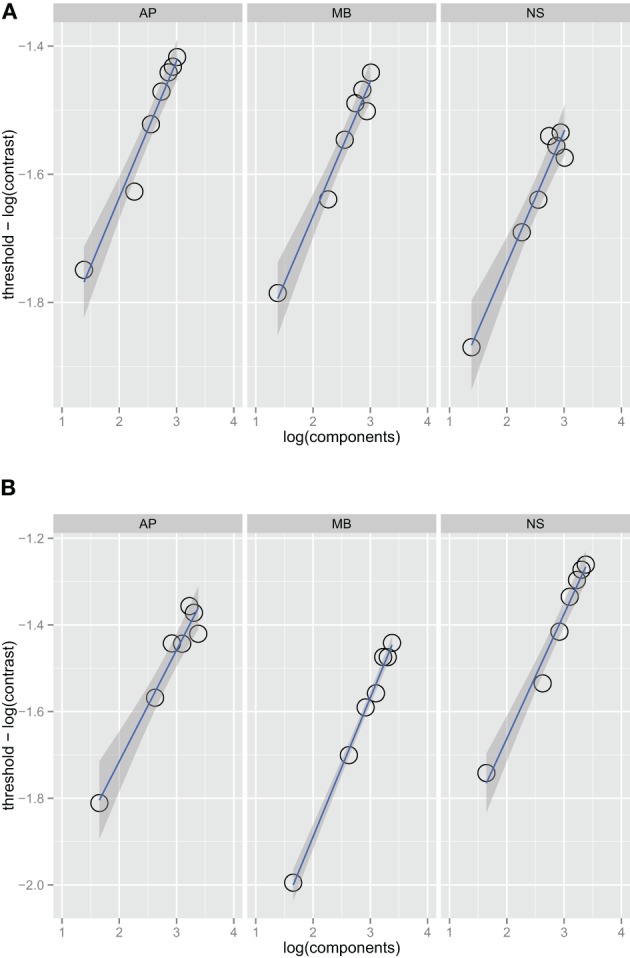
**Noise detection thresholds versus stimulus bandwidth expressed in number of spatial frequency components in the stimulus for three observers in Experiment 1 in the one octave (A) and two octave (B) spatial frequency conditions**. The blue lines are weighted maximum likelihood fits to the data and the shaded region the 95% confidence interval for the fit. The fits did not include the data from the narrowest bandwidth condition.

Figure 1 shows threshold versus bandwidth (TvB) functions for three observers in the one and two octave spatial frequency bandwidth conditions. We checked for interval effects to determine if there was a difference in threshold for stimuli presented in the first and second interval (Yeshurun et al., 2008), but there were no significant threshold differences between the two intervals. Each facet of the figure shows the thresholds for an observer (AP, MB, and NS) versus orientation bandwidth. Two-sided orientation bandwidth, which varied from 2° to 180°, is expressed as the number of Fourier components in the stimulus because the ideal observer's threshold depends on the number of components, rather than the orientation bandwidth *per se*. Figure 1 shows that detection threshold, when expressed as the logarithm of RMS contrast, increases with increasing orientation bandwidth. Each point on the graph corresponds to one of the orientation bandwidth conditions, the left-most to 2° and the right-most to 180°. There are no statistical differences between the thresholds in the one and two octave spatial frequency bandwidth conditions. The narrowest bandwidth condition was not included in the fitting procedure. If stimuli are sufficiently narrow-band then the TvB function will flatten out, producing what has been referred to in the literature as the critical-band (Quick et al., 1976). Characterizing the critical band was not the focus of this work and thus, we excluded the narrowest bandwidth, but if this point was included in the analysis, the TvB function would flatten out and have a shallower slope. Thresholds in all observers and conditions were well fitted by a power function. Bootstrap confidence intervals were calculated by simulating 999 fits to the observer data—the 95% confidence intervals always included 0.25 and ranged from 0.23 to 0.26. Finding a slope 0.25 in is line with the prediction of the quarter-root law and indicates optimal summation.

**Figure 2:**
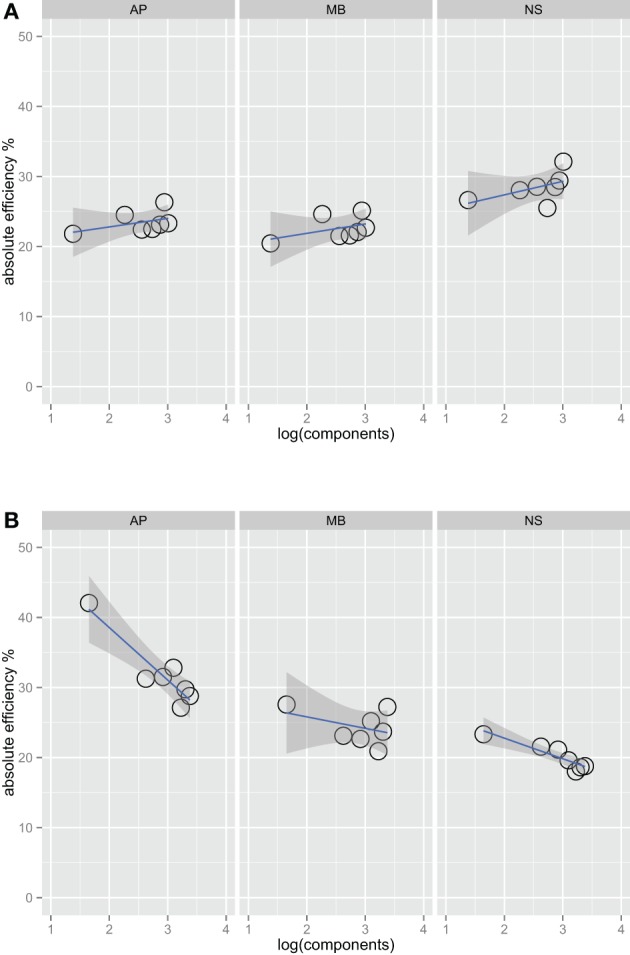
**Absolute efficiency for three observers in the one octave (A) and two octave (B) spatial frequency bandwidth conditions**. Unlike Figure 1, the fits in this plot do include the narrowest bandwidth condition. Including this point has a large effect on the efficiency versus bandwidth function for the two-octave spatial frequency condition. Observers are the most efficient at the detecting this stimulus, perhaps because it is a good match to a single component channel (Wilson et al., 1983).

Figures 2A,B show absolute efficiency (Equation 2) as a function of orientation bandwidth for the two spatial frequency conditions. For spatial frequency summation, absolute efficiency as high as 50% have been found (Taylor et al., 2009); in this experiment, absolute efficiency was also relatively high, ranging from 20% to 40%. Thresholds for the ideal observer were computed via simulations that were approximations to a two-dimensional version of that found in previous work (Kersten, 1987; Taylor et al., 2009).

**Figure 3:**
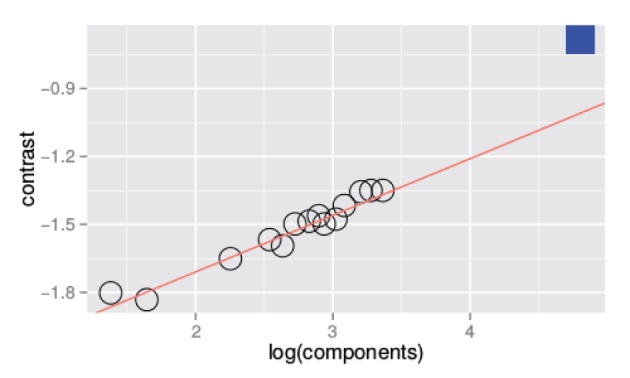
**Threshold vs. bandwidth data re-plotted from Figure 1**. Each symbol represents the average threshold from three observers and the blue square represents the average threshold for detecting unfiltered white noise. The red line is the best fitting power function with a quarter- root slope; the fit was done excluding the threshold measured with white noise.

Figure 3 is a summary figure of the data in Figure 1 and shows average detection thresholds in each spatial frequency and bandwidth condition plotted against the number of frequency components in the stimulus. The red line in the figure depicts the prediction of optimal summation for the quarter-root law. The blue square represents the mean white noise threshold, expressed in RMS contrast, for the three observers. The white noise threshold data point was not used when the combined data were fit. Including this data point provides an instructive test as it demonstrates that the quarter-root law breaks down when the stimulus includes all frequencies and orientations. Although the quarter-root law breaks down for white noise, the number of components required to observe a breakdown of the quarter-root law has yet to be determined. White noise thresholds suggest that if there is channel adjustment, there are limits to the adjustment that remain to be characterized.

The results of Experiment 1 are similar to the results for spatial frequency summation (Kersten, 1987; Taylor et al., 2009) and auditory noise detection (Green, 1960a,b) in that the TvB functions have a quarter-root slope, the same slope produced by an ideal observer. Quarter-root TvB slopes, along with the high absolute efficiencies we observed, are consistent with the idea that orientation information is summed optimally. Both of these findings are necessary but not sufficient to conclude that noise is detected by adjustable channels. As shown by our previous work on spatial-frequency summation (Taylor et al., 2009), it is important to pair estimates of threshold with the classification image method to characterize the channel used by observers. Classification images can change the interpretation of the TvB function substantially; for spatial frequency classification images lead us to interpret our data as supporting a fixed-channel model rather than an adjustable-channel model.

## 3. Experiment 2

In Experiment 2, we measured classification images with orientation filtered noise in a sub-set of conditions used in Experiment 1.

### 3.1. Materials and methods

#### 3.1.1. Observers

The two observers were 28-year old students at McMaster University who were paid for their participation. Both observers were unaware of the experimental hypotheses, had normal Snellen acuity, had extensive practice in psychophysical tasks, and participated in Experiment 1.

#### 3.1.2. Apparatus

The apparatus was identical to that used in Experiment 1.

#### 3.1.3. Stimuli

The stimuli and noise had the same parameters as those used in the one octave spatial frequency bandwidth condition in Experiment 1.

#### 3.1.4. Procedures

The procedure of Experiment 2 was the same as Experiment 1 except that the contrast of the stimulus was held constant at the 75% threshold measured in Experiment 1. There were 2500 trials per condition or classification image, for a total of 7500 trials per observer.

### 3.2. Results

We measured our classification images using a two-interval forced choice method rather than a yes/no procedure as described by Abbey et al. (1999) and calculated our classifcation images using the *power spectra* of the noise masks, rather than the noise masks themselves. This produces classifcation images in the power spectrum which has been used previously by Solomon (2002). Because the method is described in detail elsewhere, only a brief description is provided here. On each trial, the power spectrum of the noise mask in each interval was computed, the difference between the pair of power spectra calculated, and finally the difference spectrum was placed into one of four bins based on which interval contained the signal (1 or 2) and the observer's response (correct or incorrect). The difference power spectra were then averaged by the number of trials in that bin and then the two average spectra computed from correct responses were averaged, as were the two average spectra computed from incorrect trials. Finally, the difference between the correct and incorrect averaged spectra was computed and the resulting classification image was normalized to have a peak value of one. Classification images calculated using this procedure are proportional to the linear template applied to the power spectra (Abbey et al., 1999; Abbey and Eckstein, 2002).

**Figure 4:**
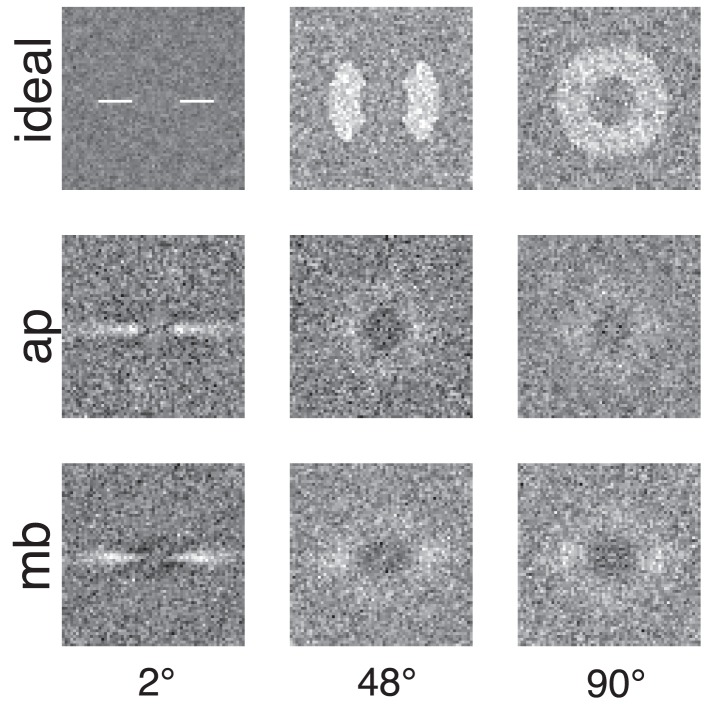
**Classification images for the ideal observer and two human observers**. Low spatial frequencies are represented by the pixels in the center of the image and frequency increases with increasing distance from the center of the image. Horizontal orientations are represented in the center row of the image and vertical orientations by the center column. All images were collected with the same number of trials. The ideal observer's classification image also serves as a depiction of filters used to generate the stimuli because the ideal observer only has access to the information in the filtered noise stimulus.

Figure 4 shows the raw classification images for the ideal observer and two human observers. Each classification image was computed using the same number of trials. The images represent spatial frequency as the distance from the center of the image. Orientation information is represented by sets of pixels in a line that begins in the center of the image and extends to its edge. The power spectra have been rotated so that the horizontal and vertical orientations in the stimulus correspond to the central horizontal row and vertical column of pixels in the image. The gray level of each pixel in the classification image represents how the power of an individual Fourier component is weighted by the observer when performing the noise detection task. If the pixel is lighter than median gray, then noise power at that frequency and orientation is positively correlated with the probability of a correct response; the lighter the pixel, the higher the correlation. Conversely, for pixels darker than median gray, power at that frequency and orientation is negatively correlated with the probability of a correct response.

**Figure 5:**
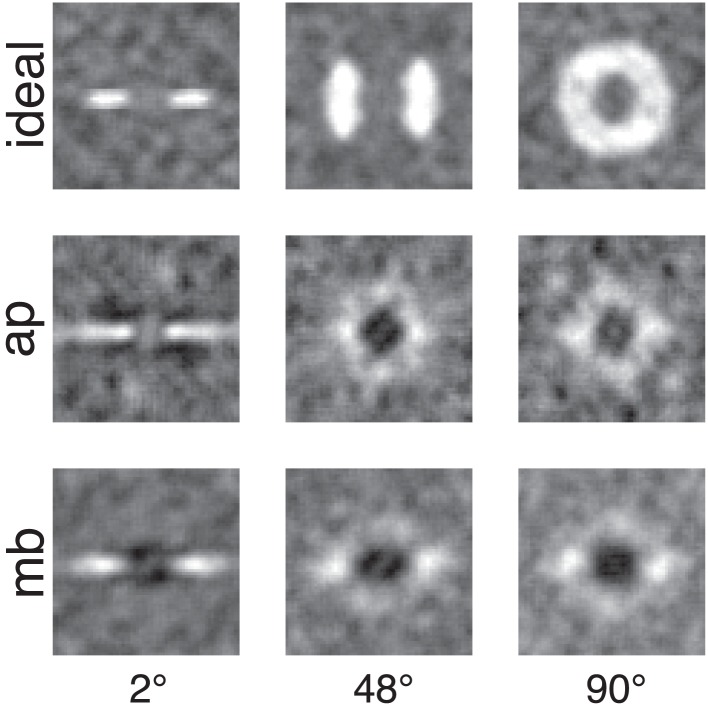
**Smoothed classification images for the same conditions and observers presented in Figure 4**. White regions represented where power in the stimulus is correlated with a correct detection response, whereas the dark or black regions indicate regions where incorrect responses are correlated with the stimulus. An important comparison to note is the difference between human and ideal observers at off-frequency and off-orientations in the stimulus. See the text for more detail.

The classification images shown in Figure 4 are 64 × 64 subsets of the full 512 × 512 power spectra which correspond to spatial frequencies from DC to approximately 20 cy/deg and include the spatial frequencies presented in the stimulus. Figure 5 shows classification images that have been smoothed with a 5 × 5 triangular convolution kernel (equivalent to linear interpolation) to reduce spurious noise in the template that results from a limited number of trials.

Figures 4, 5 show several important results. First, the human observers' classification images resemble those of the ideal observer, in that they have a narrow bandwidth (as measured by half-width at half-height) with the smallest stimulus bandwidth and get larger with increasing stimulus. Bandwidths of the classification images in the 48° and 90° conditions were larger than the bandwidths measured in the 2° condition. Also, the classification images from human observers have pronounced dark regions at off-stimulus orientations and frequencies that are not present in the classification images for the ideal observer. Noise power at these Fourier components was negatively correlated with the probability of correctly detecting the signal, an important finding that will be returned to in the discussion.

### 3.3. Analysis

**Figure 6:**
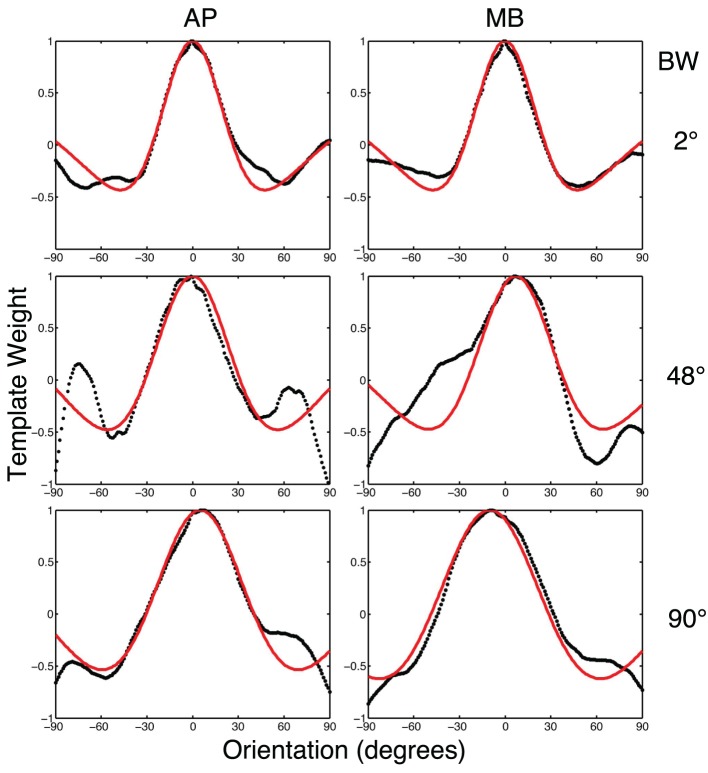
**The black dotted lines show the one-dimensional circularly summed classification images for two observers in three conditions**. The red lines show the best difference of Gaussian fits to the empirical data. The *x*-axis in each sub-figure is orientation, the *y*-axis the weight of the classification image.

To relate the classification images to orientation channels found in orientation masking experiments (e.g., Govenlock et al., 2009), the two-dimensional classification images collected in this experiment were collapsed into one-dimensional classification images as a function of orientation. Values in each classification image were summed in 1° steps across a band spatial frequencies (filter center-frequency 5 cy/deg and bandwidth of approximately 20 cy/deg) over a 180° range of orientations. The resulting values are plotted in Figure 6. Two features of the data are readily apparent. First, orientations around 0° (i.e., horizontal) had the strongest influence on observers' decisions. Second, vertical orientations or other orientations far away from zero had a weaker influence on decisions that was opposite to that of horizontal frequencies.

**Figure 7:**
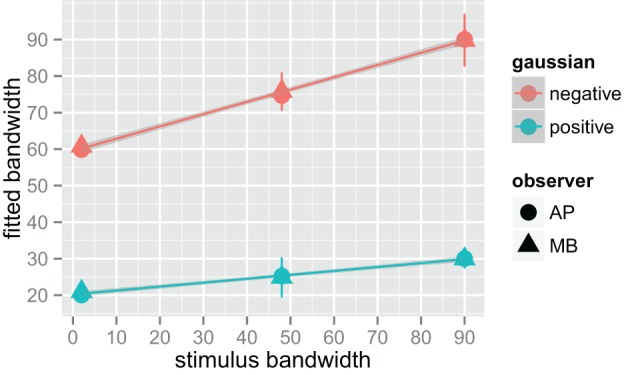
**Fitted difference of Gaussian bandwidth parameters for two observers, AP in circles and MB in triangles**. The error bars are 95% confidence intervals on the fitted parameter obtained via the bootstrap. Blue symbols are for the positive, or central Gaussian, and the red symbols the negative or surround Gaussian. R was used to obtain a weighted least squares fit to the parameters as a function of bandwidth. Both increased by roughly 50% for both observers as bandwidth was increased from the narrowest stimulus bandwidth to the widest.

We fit a Difference of Gaussians (DoG) function to our circularly summed normalized classifcation images. Classification images were normalized to the peak response. We chose DoG functions because preliminary analyses indicated that they fitted the data better than a single Gaussian and because DoG functions have been used previously to model orientation channels (De Valois et al., 1982; DeValois and DeValois, 1988; Carandini and Ringach, 1997; Ringach, 1998). We fixed the relative amplitude of the excitatory Gaussian to be twice that of the inhibitory Gaussian which is consistent with previous physiology (Sceniak et al., 2001). A DoG function has four free shape parameters—one for the center/mean and another for the bandwidth/standard deviation for each of the positive and negative Gaussians that comprise the function—but we applied two constraints that were consistent with previous models of orientation channels (Burr et al., 1981; Ringach, 1998; Shirazi, 2004). The constraints were (1) both Gaussian functions were fixed to a common center; and (2) the bandwidth of the positive Gaussian was set to be narrower than the bandwidth of the negative Gaussian. Figure 7 shows the best-fitting (least-squares) parameters and 95% confidence intervals computed via a percentile bootstrap procedure (Efron and Tibshirani, 1994). The center orientation of the best-fitting function did not change as the bandwidth of the stimulus was increased and was not different from zero, or horizontal (i.e., the orientation of the signal). The linear increase in the bandwidth for the negative Gaussian component (60–90°) was larger than the linear increase for the positive Gaussian (20–30°), although the proportional increase was about the same (i.e., 50%). Inhibitory mechanisms may be more flexible/adjustable in their responses than excitatory mechanisms. This hypotheses is supported by the data in Figure 7, specifically that slope of the red line is larger than that of the blue line.

## 4. Discussion

Our classification images support the adjustable channels hypothesis, unlike what was found in spatial frequency summation experiments (Taylor et al., 2009) using similar methods. This result implies that, contrary to the assumptions of the standard model, the mechanisms that produce optimal spatial frequency and orientation summation differ.

In the data, this point is illustrated by the negative weights in the classification images for orientation summation that occur at low spatial frequencies at all orientations but positive weights at higher spatial frequencies at a range of orientations dependent upon the signal (see Figure 5). The 1D templates derived from the 2D classification images also exhibit regions of suppression at orientations far removed from the center stimulus orientation (Figure 6), which correspond to the black/dark regions in the 2D classification images. Ideal templates do not show regions of suppression, thus the negative weights must be the result of psychological process. Furthermore, these negative weights were not found in spatial frequency summation experiments (Taylor et al., 2009), and therefore appear to be specific for orientation summation. One interpretation of these dark bands is that they reflect the contribution of inhibitory orientation processing found both psychophysically and physiologically (Ringach, 1998; Ringach et al., 2002).

The orientation bandwidths of 1D templates measured by the classification image technique become broader with increasing stimulus bandwidth (see Figure 5), but the increase in bandwidth is smaller than the channel adjustment predicted by the ideal observer. The adjustability of human observers detection mechanisms is constrained by some, as yet unknown, process. Perhaps more complex, non-linear, biologically inspired modeling (Goris et al., 2013) can capture our results, but this remains to be tested.

A possible explanation for the differences between human and ideal templates is that human observers perform the detection task by differencing the power of different spatial frequency and/or orientation components. The ideal observer knows the center spatial frequency and orientation exactly; it also knows the spatial frequency and orientation bandwidth exactly. Human observers, may not have precise access to these four signal parameters, even after many thousands of detection trials. Thus, human observers turn to an alternative strategy, one which we'll call a differencing strategy.

In Figure 5 one can see that observers use non-informative regions of the signal—anywhere the human classification image differs from the ideal classification image, this is the hallmark of the use of non-informative information (i.e., noise). Despite using non-informative information, human efficiency is still relatively high in the current task compared to efficiency in many other visual tasks (Gold et al., 1999). Why do observers use non-informative information? One hypothesis is that observers need to anchor their detection judgements and then compute a difference based on this perceptual anchor. According to this idea, observers can only make a detection decision based on the relative power within two (or perhaps more) regions of the power spectrum. In our task, the light and dark regions may represent the portions of the power spectrum that are being compared: observers may be basing their decisions on the difference between power at low spatial frequencies (at all orientations) and power at spatial frequencies and orientations within the signal band. In a given interval, if the power in the signal band is high and the power within the inhibitory region is low, observers will select that interval as the one that contains the signal. If however, the power in the inhibitory region is high and the in the signal band low, the observers will actively choose to not select that stimulus interval as containing the signal.

What is the functional role of the measured inhibitory mechanisms? One possibility, backed up by a great deal of evidence is that they play a role in contrast gain control (e.g., Watson and Solomon, 1997; Schwartz and Simoncelli, 2001). An alternative idea is that the visual system contains mechanisms that signal whether a stimulus ought to be considered an edge or a part of a texture. This hypothesis is inspired by the work on “end-stopping” found in the motion (Pack et al., 2003) and contour (Heitger et al., 1992) literature. The inhibitory mechanisms revealed by our classification images might provide a sort of end-stopping in Fourier space that limits the information that is combined into an edge or a texture. To be specific, if the orientation bandwidth within a region of visual space, as signalled by suppressive mechanisms is narrow, then it may be coded as an edge, but if the orientation content is broadly distributed then inhibitory mechanisms could provide a signal to sum orientations (and perhaps frequencies) to extract texture properties.

Work using natural images (Neri, 2014) and textures (Baker and Meese, 2014) has produced data that are broadly consistent with our results. Neri (2014) found evidence inhibition/suppression mechanisms when observers detected Gabors in noise that were either congruent/incongruent with the underlying orientation of natural scenes. He measured orientation tuning via the classification image technique and found orientation tuning and signatures of inhibitory mechanisms similar to those presented in our results (see Figure 1G). Baker and Meese (2014) used a contrast increment detection task and reverse correlation to measure the extent over which information is summed in visual space. Their reverse correlation results (see their Figures 3G,H) show the hallmarks of suppression beyond 5° of visual angle from fixation. Taken together the results above and our data provide converging lines of evidence for the use of inhibitory mechanisms that adjust tuning in orientation and visual space.

## 5. Conclusion

The goal of this paper was to determine if the results we found in our previous work on spatial frequency summation (Taylor et al., 2009) extended to orientation summation using visual noise as a stimulus. We found that detection thresholds in human and ideal observers were proportional to the quarter-root of the number of spatial Fourier components in the stimulus. Hence, orientation summation, like spatial frequency summation, was nearly optimal across a wide range of bandwidths. However, unlike what we found with spatial frequency summation, our classification image results were inconsistent with a fixed channel model. Instead, our results suggest that the orientation bandwidth of the internal filter used to detect our stimuli was adjusted to match (albeit imperfectly) the orientation bandwidth of the stimulus. The classification images also show hallmarks of inhibition at uninformative spatial frequencies and orientations and lead to the hypotheses that human observers may detect noise stimuli by comparing the power in different portions of the power spectrum.

### Conflict of interest statement

The authors declare that the research was conducted in the absence of any commercial or financial relationships that could be construed as a potential conflict of interest.
